# Preventive nebulization of mucolytic agents and bronchodilating drugs in invasively ventilated intensive care unit patients (NEBULAE): study protocol for a randomized controlled trial

**DOI:** 10.1186/s13063-015-0865-0

**Published:** 2015-09-02

**Authors:** Sophia M. van der Hoeven, Jan M. Binnekade, Corianne A. J. M. de Borgie, Frank H. Bosch, Henrik Endeman, Janneke Horn, Nicole P. Juffermans, Nardo J. M. van der Meer, Maruschka P. Merkus, Hazra S. Moeniralam, Bart van Silfhout, Mathilde Slabbekoorn, Willemke Stilma, Jan Willem Wijnhoven, Marcus J. Schultz, Frederique Paulus

**Affiliations:** Department of Intensive Care, Academic Medical Center, Meibergdreef 9, 1105 AZ Amsterdam, The Netherlands; Laboratory of Experimental Intensive Care and Anesthesiology (L E I C A), Amsterdam, The Netherlands; Clinical Research Unit, Amsterdam, The Netherlands; Department of Intensive Care, Rijnstate, Arnhem, The Netherlands; Department of Intensive Care, Onze Lieve Vrouwe Gasthuis, Amsterdam, The Netherlands; Department of Intensive Care, Amphia Hospital, Breda, Oosterhout and Etten-Leur, The Netherlands; Department of Intensive Care, Antonius Hospital, Nieuwegein, The Netherlands; Department of Intensive Care, Medical Center Haaglanden and Leidschendam, The Hague, The Netherlands; Tias/Tilburg University, Tilburg, The Netherlands

**Keywords:** Mechanical ventilation, Nebulization, Intensive care unit, Critical care, Aerosol therapy, Mucolytic agents, Bronchodilators, Acetylcysteine, Salbutamol

## Abstract

**Background:**

Preventive nebulization of mucolytic agents and bronchodilating drugs is a strategy aimed at the prevention of sputum plugging, and therefore atelectasis and pneumonia, in intubated and ventilated intensive care unit (ICU) patients. The present trial aims to compare a strategy using the preventive nebulization of acetylcysteine and salbutamol with nebulization on indication in intubated and ventilated ICU patients.

**Methods/Design:**

The preventive nebulization of mucolytic agents and bronchodilating drugs in invasively ventilated intensive care unit patients (NEBULAE) trial is a national multicenter open-label, two-armed, randomized controlled non-inferiority trial in the Netherlands. Nine hundred and fifty intubated and ventilated ICU patients with an anticipated duration of invasive ventilation of more than 24 hours will be randomly assigned to receive either a strategy consisting of preventive nebulization of acetylcysteine and salbutamol or a strategy consisting of nebulization of acetylcysteine and/or salbutamol on indication. The primary endpoint is the number of ventilator-free days and surviving on day 28. Secondary endpoints include ICU and hospital length of stay, ICU and hospital mortality, the occurrence of predefined pulmonary complications (acute respiratory distress syndrome, pneumonia, large atelectasis and pneumothorax), and the occurrence of predefined side effects of the intervention. Related healthcare costs will be estimated in a cost-benefit and budget-impact analysis.

**Discussion:**

The NEBULAE trial is the first randomized controlled trial powered to investigate whether preventive nebulization of acetylcysteine and salbutamol shortens the duration of ventilation in critically ill patients.

**Trial registration:**

NCT02159196, registered on 6 June 2014.

**Electronic supplementary material:**

The online version of this article (doi:10.1186/s13063-015-0865-0) contains supplementary material, which is available to authorized users.

## Background

Preventive nebulization of mucolytic agents and bronchodilating drugs is a strategy aimed at the prevention of airway obstruction in intubated and ventilated intensive care unit (ICU) patients [1–3]. This strategy was introduced in the 1990s [1, 2, 4], when it was recognized that invasively ventilated ICU patients are frequently unable to cough adequately, potentially resulting in stasis of sputum in the larger and smaller airways [5–7]. This could lead to clinically important atelectasis [8] and ventilator-associated pneumonia [9]. Preventive nebulization of mucolytic agents like acetylcysteine could, at least in part, solve this problem. As nebulization would dilute pulmonary secretions [10], it might be easier for a patient to evacuate secretions to the larger airways ready to be suctioned. As local application of acetylcysteine induces bronchospasm, traditionally a bronchodilating drug was added [1, 2, 11], with the additional advantage that bronchodilators like salbutamol augment mucociliary clearance [7].

Practices of nebulization during mechanical ventilation are known to be diverse, as evidence of a beneficial effect is limited and inconclusive [1, 2, 4, 12, 13]. In addition, care for the invasively ventilated ICU patient has changed significantly over the last decades. The commonly used practice of (deep) hypno-sedation and paralysis has been replaced by analgo-sedation with the restricted use of neuromuscular blocking agents [14], and ventilated ICU patients are mobilized more frequently and early during the course of their disease [15]. These changes in care may have resulted in an increased ability of invasively ventilated ICU patients to clear their airways. Consequently, this may render preventive nebulization of mucolytic agents and bronchodilating drugs an ineffective and unneeded strategy during mechanical ventilation. This may be especially true because nebulization of mucolytic agents and bronchodilating drugs carries the risk of local and systemic side effects, such as acetylcysteine-induced bronchoconstriction [1, 16, 17], and salbutamol-induced tachycardia and tachy-arrhythmias [18–20]. Also, nebulization could temporarily hamper triggering of the ventilator by a patient, potentially causing hypoventilation and patient distress [21].

The aim of the present trial is to determine the efficacy and safety of a strategy using preventive nebulization of acetylcysteine and salbutamol compared to a strategy using nebulization on indication in intubated and ventilated ICU patients. Furthermore, the related healthcare costs of this preventive strategy are determined. We hypothesize that a strategy using nebulization on indication in invasively ventilated ICU patients is not inferior with regard to the number of ventilator-free days and survival to day 28, but is associated with fewer side effects and lower health-related costs, compared to a strategy using preventive nebulization.

## Methods/Design

### Design

This randomized controlled trial investigating the (cost-) effectiveness and safety of preventive nebulization of mucolytic agents and bronchodilating drugs in intubated and ventilated ICU patients, called the NEBULAE trial, is an investigator-initiated, national, multicenter, randomized controlled, open-label, two-armed non-inferiority trial in invasively ventilated patients admitted to participating ICUs in the Netherlands.

### Trial approval and conduct

The trial is conducted according to the principles of the Declaration of Helsinki as stated in the current version of Fortaleza, Brazil, October 2013 [22], the Dutch law of Medical Research Involving Human Subjects (WMO) and Good Clinical Practice Guidelines (GCP). The trial protocol is approved by the Institutional Review Board of the Academic Medical Center, Amsterdam, The Netherlands and reviewed for local feasibility by the Institutional Review Boards of all participating medical centers represented in Additional file 1. The trial is registered at www.clinicaltrials.gov (NCT02159196) and at www.trialregister.nl (NTR4465). The trial is supported in full by a peer-reviewed grant from ZonMW, Netherlands Organization for Health Research and Development [23], which provided constructive comments on the trial design. ZonMW is not involved in the process of data collection, data analysis, data interpretation, and writing of the report. Written informed consent has to be given by the patient or their legal representative before any trial related procedure is performed. The trial will be reported according to the Consolidated Standards of Reporting Trials (CONSORT) guidelines [24].

### CONSORT diagram

**Fig. 1:**
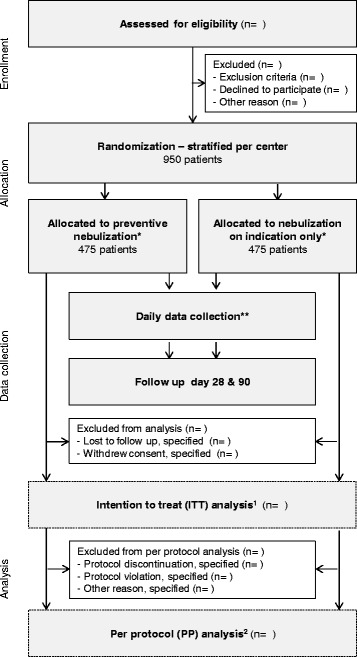
Consolidated Standards of Reporting Trials (CONSORT) diagram. *Until successful extubation; ** From day 1 until extubation, death or day 28 if still intubated, whichever comes first; ^1^Including all patients as originally allocated after randomization; ^2^Including only those patients who completed the treatment protocol as originally allocated

The CONSORT [25] diagram of the NEBULAE trial is presented in Fig. 1. Screening of consecutive patients admitted to one of the participating ICUs to verify the source population and conformance with enrolment criteria will be performed by the local investigators in all participating centers. The trial plan is to include 950 patients. If patients are excluded from participation, the reason(s) for exclusion are registered.

### Setting

The trial is performed in at least seven ICUs, one in a university-affiliated hospital (the Academic Medical Center, Amsterdam) and six in teaching hospitals (Rijnstate Hospital, Arnhem; Onze Lieve Vrouwe Gasthuis, Amsterdam; Amphia Hospital, Breda, Oosterhout and Ettenleur; Antonius Hospital, Nieuwegein; Medical Center Haaglanden, The Hague and Leidschendam; and Isala Hospital, Zwolle) in the Netherlands.

### Population

Invasively ventilated adult nonpregnant patients, aged ≥18 years, with an anticipated duration of ventilation of more than 24 hours, are eligible for participation. Patients are excluded if they received invasive ventilation in another ICU directly preceding the present ICU admission; ventilation during transport to the hospital, or ventilation started in the emergency room or in the operating theater is allowed though. In cases where the medical history necessitates the continuation of bronchodilators (for example, for chronic obstructive pulmonary disease or asthma) or in cases of a known allergy to the trial medication, the patient will be excluded from participation. Patients with neuromuscular diseases or complete spinal cord lesions, and consequently with an expected need for long-term ventilation, are also excluded. Finally, previous randomization in the NEBULAE trial excludes patients from participation as well.

### Patient enrollment, randomization and blinding

Consecutive patients are screened for eligibility by local investigators and treating physicians. Patients meeting all inclusion criteria and no exclusion criteria are candidates for inclusion in the study and will be randomly assigned in a 1 to 1 ratio, after informed consent is given by the patient or their legal representative, to either a strategy of preventive nebulization of acetylcysteine and salbutamol four times daily (the ‘preventive nebulization’ arm) or nebulization of acetylcysteine or salbutamol on indication (the ‘nebulization on indication’ arm). Randomization will be performed by the local investigators and treating physicians as soon as possible, but always within 24 hours after start of invasive ventilation in the ICU. To avoid prediction of future patients’ allocation, randomization sequence is generated using a permuted block design with random block sizes stratified by study center. Maintenance of allocation concealment will be warranted using a central, dedicated, password-protected, SSL-encrypted web-based, automated randomization system, available 24 hours per day (ALEA® software, TenALEA consortium, Amsterdam, The Netherlands), developed by the Clinical Research Unit in the Academic Medical Center, Amsterdam, The Netherlands, which is not involved in the conduct of the study. Due to the nature of the intervention, blinding of the patients and caregivers is not possible. Outcomes assessment will be performed according to the study protocol by independent ICU physicians not involved in the trial. Investigators performing analysis are blinded for the intervention.

The assigned strategy is continued until tracheal extubation. If a patient requires reintubation and additional invasive ventilation within a period of 28 days, the strategy to which the patient is randomized will be resumed.

### Interventions

When randomized to the preventive nebulization arm, the patient will receive nebulization every 6 hours with solutions containing 300 mg acetylcysteine *plus* 2.5 mg salbutamol. Flasks of acetylcysteine and salbutamol are provided by the pharmacy of the participating center. Since no placebo will be used, labeling of the drugs is not necessary. Solutions are nebulized using jet nebulizers or vibrating mesh nebulizers, depending on local availability in the participating centers. Nebulizers are set according to manufacturer’s recommendations. External nebulizers are powered by pressured air or oxygen at a continuous flow of 3 to 5 L/min. The nebulizer will be attached to the tubing system, always after the heated humidifier or the heat and moisture exchange (HME) filter (that is, at the side of the patient). A nebulization session typically lasts 20 to 30 minutes. The type of nebulizer used will be recorded in the case record form (CRF).

When randomized to the nebulization on indication arm, nebulization of acetylcysteine *or* salbutamol is restricted. The doctor staff can decide to order nebulization of solutions containing 300 mg acetylcysteine *or* 2.5 mg salbutamol in case a patient has persistent thick and tenacious secretions [26] or in cases of bronchospasm (suspected when there is a clinical wheezing, and signs of obstruction of the lower airways on ventilator waves or the end-tidal CO_2_ curve), respectively. Daily reassessment of the indications mentioned above will reassure that nebulization is stopped when no longer needed. The decision to start and stop nebulization is recorded in the CRF.

### Concomitant medications

Mucolytic agents or bronchodilating drugs other than acetylcysteine and salbutamol are not used. Nebulization of drugs other than mucolytic agents or bronchodilating drugs (for example, antimicrobial and antimycotic agents, or ilomedine and iloprost) however, remains possible. They should be administered separately from the nebulization of acetylcysteine and salbutamol and recorded in the CRF.

### Standard lung protective care

Standard care follows the local clinical guidelines and is performed by independent ICU physicians and nurses not involved in the trial. Doctor and nursing staff are encouraged to use lung-protective ventilation strategies, including the use of lower tidal volumes; restrictive sedation; early weaning, preferably by the use of weaning trials; fluid restriction; and infection prevention.

Lung-protection includes the use of lower tidal volumes (6 ml/kg predicted body weight, in patients with ARDS [27, 28], as well as in patients without ARDS [29]); higher levels of positive end-expiratory pressures (>5 cm H_2_O) are only used in cases of suspected shunting or signs of moderate or severe ARDS [30, 31].

Restrictive sedation includes the use of a local sedation guideline aiming at analgo-sedation and preventing hypno-sedation [32, 33], with a preference for intermittent sedation over continuous infusion of sedatives [34]; sedation depth is evaluated at least three times a day using the Richmond Agitation Sedation Scale (RASS) [35, 36]; pain is scored using the Numeric Rating Scale (NRS), Visual Analog Scale (VAS), Critical Care Pain Observation Tool (CCPOT) or Behavioral Pain Scale (BPS), and treated accordingly [37–39]. Delirium is assessed at least three times daily using a standardized tool, that is, the Confusion Assessment Method for the ICU (CAM-ICU) or the Intensive Care Delirium Checklist Screening (ICDCS) [40].

Doctor and nursing staff are encouraged to frequently test whether a patient triggers the ventilator and, if so, to use spontaneous modes of ventilation. Readiness to wean will be assessed on a daily basis. Pressure–support level is lowered stepwise to 5 cm H_2_O, as soon as patients are ready to be weaned from the ventilator. Attending physicians will decide to extubate the patient, based on general extubation criteria (for example, patient is responsive and cooperative with an adequate cough reflex, an adequate oxygenation saturation (>90 %) with PaO2/FiO2 of >200 mmHg and FiO2 ≤40 % and a respiratory rate of 8 to 30 per minute with no signs of respiratory distress (that is, marked accessory muscle use, abdominal paradox, diaphoresis, or marked dyspnea), is hemodynamically stable with no uncontrolled arrhythmia, and has a rectal temperature >36.0°C).

Fluid management follows the local guidelines and is dictated by clinical needs but aims at a neutral fluid balance as soon as possible after stabilization and weaning of inotropes and/or vasopressors [41]. Crystalloid infusions are favored over colloid infusions [42]. Transfusion of blood products is restricted.

Infection prevention consists of oral care, combining tooth brushing and rinsing of the oral cavity every 6 hours, and selective oropharyngeal decontamination or selective decontamination of the digestive tract are applied to all patients according to local clinical guidelines to prevent nosocomial infections [43]. Head-of-bed elevation is pursued [44]. Ventilator circuits are not changed routinely unless a circuit is visibly contaminated or damaged [45].

### Standard airway care

Independent ICU physicians and nurses not involved in the trial perform standard airway care, including endotracheal suctioning, humidification of inhaled air and tracheostomy placement.

Endotracheal suction is performed when clinically indicated and according to the local guidelines [46]. Normal saline instillation prior to endotracheal suction will not be used [46]. The use of an open or closed endotracheal suction system is left to the discretion of the participating center and will be recorded in the CRF.

Humidification and heating method of the inhaled air is left to the discretion of the participating center and will be recorded in the CRF. Active humidification of the air inhaled will be supplied by the use of an electrically powered humidifier. Passive humidification of the air inhaled will be supplied by the use of a heat and moisture exchange filter.

Tracheostomy is considered on indications only and preferably not in the first ten days after intubation [47]. Indications for tracheostomy are failure to intubate, expected duration of ventilation >14 days, Glasgow Coma Score <7 and/or inadequate swallow or cough reflex with retention of sputum, severe ICU acquired weakness, prolonged or unsuccessful weaning and repeated respiratory failure after tracheal extubation.

### Primary outcome and secondary outcomes of the trial

The primary outcome of this study is the number of ventilator-free days (VFD) and survival to day 28, defined as the number of calendar days of unassisted breathing, after both ICU admission and start of mechanical ventilation to day 28, if the period of unassisted breathing lasted at least 24 consecutive hours. Unassisted breathing is defined as being extubated, ventilating with or without facemask, nasal prong oxygen, room air or tracheostomy breathing, but always without continuous positive airway pressure or pressure support of intermittent mandatory ventilation assistance. Patients who die before 28 days or are mechanically ventilated longer than 28 days are assigned zero ventilator-free days.

Secondary outcomes include clinical endpoints and cost-effectiveness outcomes. Clinical endpoints include the following: ICU and hospital length of stay (LOS); ICU, hospital, and 90-day mortality; development of moderate or severe ARDS; ventilator-associated pneumonia (VAP); development of atelectasis and pneumothorax; incidence of obstruction of the endotracheal tube (wherefore endotracheal tube exchange is indispensable); cumulative dose and duration of sedatives, cumulative dose and duration of neuromuscular blocking agents, development of delirium and incidence of side effects of nebulization of acetylcysteine and/or salbutamol. In addition, related healthcare costs will be compared from a health systems perspective over the time horizon of the trial and include direct medical and indirect costs of days of mechanical ventilation, stay in the ICU and hospital, cumulative use of sedatives and neuromuscular blocking agents, tracheostomies and ventilator-associated pneumonia.

### Clinical data to be collected

Data on baseline, demographic characteristics and disease severity are collected on the day of enrolment, including gender, age, height, weight, reason for ICU admission, reason for invasive mechanical ventilation and the APACHE II score. Furthermore, data are collected on the medical history including presence of diabetes mellitus, severe heart failure (New York Heart Association NYHA classification) [48], acute coronary syndrome or pulmonary disease, pulmonary or hematological malignancies, previous pulmonary radiation and/or surgery, and current use of immunosuppressive medication.

Data on clinical outcomes are collected on a daily basis up to day 28, discharge of the ICU or death, including data on duration of ventilation, length of stay in ICU and hospital. Location of the patient (ICU, hospital, other facility, or home) and life status (alive or deceased) is assessed on days 28 and 90.

Clinical data are collected on a daily basis up to day 28, discharge from the ICU or death, and these data include: intubation status (if extubated: time of extubation), tracheostomy status, mechanical ventilation parameters (see below), development of pulmonary complications (moderate or severe ARDS, pneumonia, atelectasis and pneumothorax); cumulative use and duration of sedatives and neuromuscular blocking agents; sedation score using the Richmond Agitation Sedation Scale (RASS) [35, 36]; level of pain (Numeric Rating Scale or Visual Analog Scale or Critical Care Pain Observation Tool or Behavioral Pain Scale) [37–39]; delirium score with the Confusion Assessment Method for ICU (CAM-ICU) or Intensive Care Delirium Checklist Screening (ICDCS) [40] score; amount and type of infused blood products; cumulative fluid balance and Sequential Organ Failure Assessment (SOFA) [49].

Parameters of mechanical ventilation are collected 1 hour after intubation and every day at a fixed time point up until extubation, day 28 or death including the following: tidal volume, respiratory rate, levels of PEEP, Ppeak and/or Pplateau, the level of pressure support, inspiration to expiration ratio, oxygen fraction of inspired air, minute volume and pulmonary compliance. The Lung Injury Score [50] and Oxygenation Index [51] are calculated.

Compliance data are collected on a daily basis until extubation up to day 28 or death, including the quantity of secretions, type of humidifier (active or passive), amount and indication of nebulization of acetylcysteine and/or salbutamol, episodes of nebulization of other agents and side effects of nebulization of acetylcysteïne and/or salbutamol.

Resource-use parameters and unit prices are collected to estimate healthcare costs from a health systems perspective and include the medical costs of ventilation, ICU stay, in hospital stay, cumulative use of sedatives and neuromuscular blocking agents, tracheostomies and ventilator-associated pneumonia.

### Sample size calculation

Randomized trials assessing the efficacy of preventive nebulization are absent, which hampered power calculations. Therefore, the sample size calculation, based on a non-inferiority design, is calculated using the mean duration of invasive ventilation and the associated coefficient of variation, respectively 5 and 0.7 (log-transformed data) days [52], with a noninferiority margin of 10 %. A maximum difference in clinical effectiveness of 10 % between the ‘nebulization on indication’ arm as compared to the ‘preventive nebulization’ arm will be tolerated to consider a strategy of ‘nebulization on indication’ not to be inferior, with regard to the primary endpoint ventilator free days. Using these assumptions associated with the study design, a one-sided type I error of 0.05 and 80 % power (type II error), yields a sample size of 950 patients, which includes a 5 % anticipated drop out.

### Interim analysis

Interim analyses regarding safety are performed by an independent Data Safety Monitoring Board (DSMB) when 317 and 634 patients, have been included and have completed follow-up for the primary outcome. These interim analyses are not related to the non-inferiority hypothesis.

### Data analysis

The main analysis will compare the number of ventilator-free days and survival to day 28 between the two randomized groups. If the non inferiority of nebulization on indication compared to preventive nebulization can be confirmed, the superiority of nebulization on indication compared to preventive nebulization for the primary endpoint will be tested.

In addition, we will analyze the non inferiority between the two treatment groups regarding their survival distributions. Considering that survival times are not normally distributed and because some survival times will be censored by mortality, the Cox proportional-hazards model will be used to analyze the data. Significance will be expressed by the standard log rank test.

The effect of preventive nebulization on the primary outcome will also be investigated in prespecified subgroups based on patient categories (namely, patients with pneumonia versus patients without pneumonia and surgical versus medical admissions), and will be adjusted for the type of nebulizer (jet nebulizer or vibrating mesh), humidification method (active or passive), and type of flow used for nebulization (continues with external source or breath synchronized with an inline flow from the ventilator).

### General statistical considerations

Initial analyses will be performed according to the intention-to-treat (ITT) method. In addition, per-protocol (PP) analysis, including only those patients who complete the treatment according to the originally allocated protocol, will be done to check for the robustness of the results.

Continuous normally distributed variables will be expressed by their mean and standard deviation, or when not normally distributed as medians and their interquartile ranges. Categorical variables will be expressed as counts (n) and percentages (%). To analyze differences in continuous variables between the two groups, Student’s t-test will be used, or in case continuous data is not normally distributed, the Mann–Whitney U test will be used. Categorical variables will be compared with the Chi-square test or Fisher's exact tests. Statistical significance is considered to be at a *P* value <0.05 with one or two side test, depending on assessment of either non inferiority or superiority. The ICU mortality, hospital mortality, the length of ICU stay, and the length of hospital stay will be expressed with Kaplan-Meier curves.

When appropriate, statistical uncertainty will be expressed by 95 % confidence levels. All statistical analyses will be performed with the R language and environment for statistical computing [53].

### Economic evaluation

Alongside the proposed RCT, a prospective economic study will be performed. The main question in the economic evaluation is whether the beneficial effects of preventive nebulization, compared to nebulization on indication, justifies the healthcare costs of preventive nebulization. This analysis is set up as a cost-benefit analysis (CBA), incorporating costs that will be estimated from a health system perspective over the time horizon of this study. In this economic evaluation, costs will be determined for both randomization groups during a 28-days follow-up period after ICU admission and start of mechanical ventilation. The costs of medical care will be calculated, divided into direct medical, direct nonmedical, and indirect costs, if applicable. Costs are defined as the volumes of used resources multiplied by the calculated unit prices. No discounting of costs and effects will be applied. Although, if any difference of interest in primary and/or secondary outcomes is observed, registered resource use and related costs will be compared between both intervention groups. In addition a cost-effectiveness analysis, to calculate incremental cost ratios (ICER), will be performed.

### Study organization

Trial oversight will be provided by a Trial Steering Committee composed of the principal investigators, the coordinating investigator and local investigators in the participating centers. The principal investigators and the trial coordinator are responsible for daily management of the trial. They provide assistance to the participating sites in trial management, record keeping, data management and training of the local staff. Local investigators in each site will perform randomization, supervise data collection and ensure adherence to the ICH-GCP guidelines during the trial.

### Data management

All data are coded by a patient identification number (PIN). The key is kept at the trial sites in a secure place. The data are transcribed by the local investigator into a central GCP-proof internet-based electronic CRF. Recorded data, provided with a code, will be stored securely for 15 years in archives of the Academic Medical Center, Amsterdam, The Netherlands. Data will be accessible only by the principal investigators and representatives of the Inspectorate for Healthcare of The Netherlands.

### Monitoring, safety and the reporting of adverse events

An independent GCP-certified monitor will monitor the study for data quality according to the ICH-GCP guidelines. During onsite visits, monitoring will be conducted on the following: progress of the study, rights and wellbeing of the subjects, completeness and accuracy of the recorded data, verifiability of the recorded data from source documents, compliance with the approved protocol and amendments, and compliance with GCP and applicable national regulatory requirements. Every participating center will be visited at least once every year.

Monitoring of patient safety and reviews of safety issues will be performed by a designated independent DSMB. The DSMB, consisting of three experts of critical care and mechanical ventilation and an independent statistician, watches over the ethics of the conduct of the study in accordance with the Declaration of Helsinki. The DSMB will meet by conference calls. Meetings are scheduled prior to the first patient enrolment, after inclusion of respectively 317 and 634 patients when the interim analyses for safety are performed, and after completion of patient enrolment.

As the study population consists of critically ill patients, we expect many of the patients to develop serious adverse events according to the GCP guidelines’ definition. Serious adverse events (SAEs) considered (possibly) related to the study procedure according to the local investigator will be reported to the study coordinator, SAE manager and principal investigator within 24 hours. If at least one or both (that is, the SAE manager and/or principal investigator) judges the association with the study intervention as (possibly) related, the SAE is classified as (possibly) related and reported to the reviewing Institutional Review Board and DSMB within 15 days. All (S)AEs will be provided to the reviewing Institutional Review Board and DSMB in a line listing format every 6 months. If complications occur significantly more often in the intervention group, termination of the study due to harm can be considered by the DSMB. Should the principal investigator decide not to fully implement the advice of the DSMB, the principal investigator will send the advice to the reviewing Institutional Review Board, including a note to substantiate why (part of) the advice of the DSMB will not be followed.

## Discussion

Mechanical ventilation is associated with an impaired clearance and retention of airway secretions, which can lead to airway obstruction, atelectasis, bacterial colonization and pulmonary infection [2, 7, 54]. Preventive nebulization of mucolytic agents and bronchodilating drugs is a strategy suggested to prevent these complications [2, 4, 7]. No previous studies have addressed whether this preventive strategy is effective in a general ICU population of intubated and ventilated ICU patients.

NEBULAE is the first randomized controlled trial sufficiently powered to investigate the effectiveness, safety and costs of preventive nebulization of mucolytic agents and bronchodilating drugs compared to nebulization on indication.

All patients with an expected duration of ventilation of more than 24 hours are eligible for inclusion. Patients are excluded if they are admitted and invasively ventilated in another hospital before admission to be able to determine the total duration of ventilation and guarantee recording of all episodes of nebulization.

A concern regarding safety could be the occurrence of endotracheal tube occlusion, although nebulization of acetylcysteine is allowed in both groups in case of thick and tenacious pulmonary secretions. Furthermore, nebulization of liquids and solutions may cause obstruction and malfunction of the expiratory valve due to deposition of the aerosolized medication. To minimize this risk, awareness and training about this risk among nurses and physicians is advocated. Thereby, filters will be used and changed daily to protect the ventilator. An interim analysis on safety will be performed to evaluate these concerns. Furthermore, sedation practice could mask potential patient discomfort caused by the intervention. Similar sedation aims in both arms of the trial need to be persuaded.

The main strength of this study is the potential generalizability of its findings, considering the participation of different types of ICU’s in several hospitals as well as the broad spectrum of patients being eligible for participation. Besides the intervention under study, which is a well-known procedure familiar to nurses and physicians in all participating centers, no additional measurements or procedures are part of the study protocol. Therefore, execution of the study is straightforward and feasible.

This trial also has some weaknesses: first, due to the nature of the intervention, blinding of patients and caregivers is not possible. Acetylcysteine has a very typical odor that cannot be mimicked, and consequently, nebulization with saline as placebo is not possible. Investigators who perform the analyses however are blinded for the intervention. In addition, the weaning process that directly, influences the primary outcome, as well as the airway care, will be performed by the attending ICU physician and nurse not involved in this study. Second, despite the fact that indications for nebulization of mucolytic agents or bronchodilating drugs in the ‘the preventive nebulization arm’ are set in the study protocol, indications are known to be highly interpretable. Third, as this is a multicenter study, differences in standard practice between participating centers exist. The study protocol, however, stresses that standard care be performed according to internationally accepted guidelines. Moreover, the randomization to both study arms is stratified per center.

Participating centers are allowed to administer humidification according to their local standard practice. Nebulization of mucolytic agents and bronchodilating drugs will be applied using either a jet nebulizer, the most commonly used type of nebulizer [13] or a vibrating mesh nebulizer. The type of nebulizer, as well as the type of humidification, are suggested to influence the fraction of nebulized medication reaching the lung [55, 56]. As a consequence, uniformity of the studied intervention might be diminished. This choice was made since we aim to study current nebulization practice and intent to avoid additional risks by introducing a new procedure with potential serious adverse outcomes. To control for these factors, randomization is stratified per center. In addition, data on applied care and on the use of relevant concomitant strategies possibly influencing outcomes are collected. Also, prespecified subgroup analyses are performed.

In conclusion, the NEBULAE trial investigates the effectiveness, safety and health-related costs of preventive nebulization of mucolytic agents and bronchodilating drugs compared to nebulization of mycolitic agents and/or bronchodilating drugs on indication only, in intubated and ventilated ICU patients, with regard to the number of ventilator-free days and survival to day 28. Results of this trial will guide local and international practice guidelines regarding care for the invasively ventilated patient.

## Trial status

Patient recruitment for the NEBULAE trial is currently ongoing in seven Dutch hospitals. Enrollment started on 22 July 2014. The anticipated recruitment completion day is 1 June 2016.

## Additional file

Additional file 1:
**Names of the Institutional Review Boards that have approved the NEBULAE study in the participating centers.** (DOC 23 kb)

## Abbreviations

AE: adverse event
ALI: acute lung injury
ARDS: acute respiratory distress syndrome
BPS: Behavioral Pain Scale
CCPOT: Critical Care Pain Observation Tool
CONSORT: Consolidated Standards of Reporting Trials
COPD: chronic obstructive pulmonary disease
CRF: case record form
DSMB: Data Safety and Monitoring Board
GCP: good clinical practice
HME: heat and moisture exchanger
ICU: intensive care unit
I:E: inspiratory to expiratory ratio
LIS: Lung Injury Score
LOS: length of stay
NRS: Numeric Rating Scale
PBW: predicted body weight
PEEP: positive end-expiratory pressure
PIN: patient identification number
RASS: Richmond Agitation Sedation Scale
SAE: serious adverse event
SOFA: Sequential Organ Failure Assessment
SpO2: oxyhemoglobin saturation by pulse-oximetry breathing air in supine position
VAP: ventilator associated pneumonia
VAS: Visual Analog Scale
VFD: ventilator-free days
WBC: white blood cells
