# Supplementary material for: Preventive nebulization of mucolytic agents and bronchodilating drugs in invasively ventilated intensive care unit patients (NEBULAE): study protocol for a randomized controlled trial
Source: Trials. 2015 Sep 2;16:389. doi: 10.1186/s13063-015-0865-0 (PMC4557315; doi:10.1186/s13063-015-0865-0)
Supplement: Additional file 1: — Names of the Institutional Review Boards that have approved the NEBULAE study in the participating centers. (DOC 23 kb) [file 13063_2015_865_MOESM1_ESM.doc]

**Appendix 1. Names of institutional reviewing boards approving the NEBULAE study in the participating centers:**

Institutional Review Board of the Academic Medical Center, Amsterdam, The Netherlands (reference number: 2014_088)

Local Feasibility Committee Rijnstate Hospital, Arnhem, The Netherlands (reference number: LTC 1029/110714)

Medical Ethical Committee Onze Lieve Vrouwe Gasthuis, Amsterdam, The Netherlands (reference number: WO 14.050)

Ethical Committee Human Research Amphia Hospital, Breda, Oosterhout and Ettenleur, The Netherlands (reference number: OS/FdH/IN/14.0481//7.25)

Department of Research & Development Antonius Hospital, Nieuwegein, The Netherlands (reference number: R&D/L14.040/Nebulae)

Local Feasibility Committee Medical Center Haaglanden, The Hague and Leidschendam, The Netherlands (reference number: 2014-054)

Medical Ethical Review Committee Isala Hospital, Zwolle, The Netherlands (reference number: 15.0232)
